# Rules for Dyspeptics

**Published:** 1891-11

**Authors:** 


### RULES FOR DYSPEPTICS.

Dyspeptics will do well to observe the following general rules :

Live on two meals a day, if possible. Never eat to excess. Estimate as nearly as you can the actual needs of the system, and limit the quantity of food to them, remembering that one grows weak if he eats too much. Eat slowly and masticate all food even longer and more thoroughly than a healthy person careful of his digestion would do. Quite dry foods, as a rule, are best suited to dyspeptics, who should drink sparingly with their meals. Some can take ice water in very small quantities without being disturbed by it, but generally it retards digestion. And the same is true of all cold drinks. Warm ones suit most dyspeptics best ; and a very little weak tea, if properly made, is not at all likely to do any harm. The food should be neither very hot nor very cold. Properly, it should be about "blood warm." Every one knows from experience just what foods distress them and what are well borne ; of course, the former should be excluded from the diet. There are but few people who cannot eat good ripe fruits in season, provided they do so in the morning. Fruits, raw or cooked, if eaten with the supper, often cause digestive disturbances.

After dinner rest awhile and the rest should be mental as well as physical. But in the course of an hour or two after eating, most dyspeptics are benefited by a walk of a mile or two, the distance being covered leisurely. This is usually the case if what is known as flatulent dyspepsia is suffered from. During the walk the "fullness" disappears and the victim of it returns feeling buoyant, physically as well as mentally.

Some dyspeptics are very thirsty a little while after eating heartily. Hot water is the best drink for them, and it should be sipped. This is also a remedy for "sour stomach," and many other symptoms which come under the head of "distress after eating." When one is very tired mentally or physically, the digestive organs share in the fatigue, and they ought to have a short rest before being put to work. If a feeling of faintness or "goneness" exists and urges one to eat at once while tired and worried, he should slowly sip a cup of hot water, and sit down or lie down, and occupy himself for fifteen or twenty minutes with a newspaper or novel. In that way he can comfortably put up with the delay. A ruffled temper always obstructs digestion, and the table should be sacred from the intrusion of discussions at all likely to cause dissension.—*Ex.*

